# Effectiveness of community-based treatment programs for treatment of uncomplicated severe acute malnourished children aged 6–59 months using locally produced nutrient dense foods: protocol for a multicentric longitudinal quasi-experimental study

**DOI:** 10.1186/s40795-021-00489-1

**Published:** 2021-12-15

**Authors:** Praveen Kumar, Rajesh Kumar Sinha, Abner Daniel, Hemang Shah, Raja Sriswan, Arun Kokane, Aditya Mohapatra, Vivek Kashyap, Anil Kumar Goel, Virendra Kumar, Asha Kiran, N. Arlappa, Ankur Joshi, Rashmi Ranjan Nayak, Manjula Singh, Mihretab Salasibew, Samik Ghosh, Sameer Manikrao Pawar, Preetu Mishra, Khyati Tiwari, Sourav Bhattacharjee, Farhat Saiyed, Tarun Shrikrishna Patel, Pritish Kumar Nayak, Sanjay Kumar Sahoo, Mahendra Prajapati, Shikha Sinha, Arjan de Wagt

**Affiliations:** 1 Lady Hardinge Medical College and associated Kalawati Saran Children’s Hospital, C-604 Connaught Circus, DIZ Area, Connaught Place, New Delhi, 110001 India; 2 National Centre of Excellence for Management of Children with Severe Acute Malnutrition (NCoE-SAM), Kalawati Saran Children’s Hospital, C-604 Connaught Circus, DIZ Area, Connaught Place, New Delhi, 110001 India; 3 UNICEF India Country Office, 73, Lodi Estate, New Delhi, 110003 India; 4 Children’s Investment Fund Foundation, The Crescent, Level 3, Lado Sarai, New Delhi, 110030 India; 5 ICMR-National Institute of Nutrition, Beside Tarnaka Metro Station, Osmania University, PO, Hyderabad, Telangana 500007 India; 6 All India Institute of Medical Sciences, Saket Nagar, AIIMS Campus, Saket Nagar, BaghSwaniya, Bhopal, Madhya Pradesh 462020 India; 7 Annex Building, SIHFW, BiraMaharana Ln, Nilakantha Nagar, Nayapalli, Bhubaneswar, Odisha 751012 India; 8 Rajendra Institute of Medical Sciences, Ranchi, Jharkhand 834001 India; 9 AIIMS Campus, Gate No, 1, Great Eastern Rd, opposite Gurudwara, Tatibandh, Raipur, Chhattisgarh 492099 India; 10 Department of Women and Child Development and Mission Shakti, Government of Odisha, Mission Shakti Bhawan, At-Gandamunda, PO-Baramunda, Bhubaneswar, Odisha Pin-751030 India; 11 UNICEF, Plot No.41-42, Polytechnic Colony, Shyamla Hills, Bhopal, Madhya Pradesh 462013 India; 12 UNICEF, VISHWA Complex, Ground Floor, Near IICM, Kanke Road, Ranchi, Jharkhand 834006 India; 13 UNICEF, Flat No. 1104, Block B, Indis One City, KPHB, Hyderabad, Telangana 500072 India; 14 UNICEF, 44 Surya Nagar, Bhubaneswar, Odisha 751003 India; 15 UNICEF, Chhattisgarh, India

**Keywords:** Community based Management of Acute Malnutrition, Severe acute malnutrition, Locally produced nutrient dense food supplements, Cure rate, Body composition

## Abstract

**Background:**

Severe acute malnutrition (SAM) is a major underlying cause of mortality among children. Around one third of the world’s acutely malnourished children live in India. The WHO recommends community-based management of acute malnutrition (CMAM) for managing children with SAM. In India, different states are implementing community-based SAM treatment programme, hereinafter called CSAM, using varieties of locally produced nutrient dense food items with different nutrient compositions. The study will assess the effectiveness of these state specific CSAM interventions.

**Methods:**

The longitudinal quasi-experimental study will be undertaken in two purposively selected blocks of one district each in the four intervention states and one comparison state. From each state, 200 SAM children identified using weight-for-length/height z-score (WHZ) < **−** 3 criteria will be enrolled in the study. Their anthropometric data and skinfold thickness will be taken on admission, at sixth week and at discharge by trained field investigators. Other child details, incidence of morbidity and socio-economic details will be collected on admission. To assess food consumption pattern including consumption of locally produced nutrient dense food supplements, dietary assessment, using 24-**h** dietary recall will be conducted on admission, at sixth week and at discharge. In addition, body composition parameters will be assessed for a sub-set of children using bio-electrical impedance analysis on admission and at discharge to analyse changes in total body water, fat-free mass, and fat mass. Post discharge, all study participants will be followed up monthly until 6 months. Atleast 10% of the sample will be checked for quality assessment.

The study’s primary outcome is cure rate defined as children attaining WHZ **≥** -2. Secondary outcomes include mean weight gain, mean length of stay, body composition parameters, relapse and mortality rates. Additionally, process evaluation and cost effectiveness analysis will be conducted.

**Discussion:**

There is a shortage of robust evidence regarding the effectiveness of locally produced nutrient dense food supplements provided as part of the CSAM intervention in India. This study will contribute to evidence on effective strategies to manage children with uncomplicated SAM in India. The study protocol has all necessary ethical approvals. Written informed consent will be obtained from caregivers of the children.

**Trial registration:**

The study is registered with Clinical Trial Registration of India (Registration No.: CTRI/2020/09/028013) Date of registration 24/09/2020.

**Supplementary Information:**

The online version contains supplementary material available at 10.1186/s40795-021-00489-1.

## Background

Severe Acute Malnutrition (SAM) is defined as a weight-for-height/weight-for-length z-score < − 3 (WHZ < -3) and/or mid upper arm circumference (MUAC) < 115 mm and/or clinical signs of bilateral pitting edema in children aged 6–59 months [1]. Globally, approximately 50 million under-five children suffer from wasting (WHZ < -2). India is home to approximately 18.3 million wasted children, representing 37% of the global burden of wasting. Of this, approximately 8 million children are severely wasted (WHZ < -3) [2]. The two national surveys have shown worsening of child wasting and severe wasting conditions in India [3, 4]. A recent pooled-analysis of existing community cohort studies has shown that mortality risk in severely wasted children was 11.6 times higher than in children without severely wasted [5]. In India, evidence also shows that around 20% of all deaths of children aged under-five years were due to wasting [6]. Other studies conducted in Bangladesh [7] and Latin America [8] have also found strong nutrition-mortality linkage among under-five children. Early identification and treatment before development of medical complications is essential to prevent mortality among SAM children.

The World Health Organization (WHO) recommends community-based management of acute malnutrition (CMAM) for early identification and treatment of children with SAM with no medical complications. CMAM includes community outreach for screening of children for SAM, outpatient management of uncomplicated SAM children through provision of ready-to-use therapeutic food (RUTF) and antibiotics, and in-patient treatment of medically complicated SAM children [9]. Evidence shows that community-based treatment program identifies and treats uncomplicated SAM children much before they develop any medical complications [10]. It also provides higher coverage through a regular community based screening process and complete treatment by enabling the child to be fully cured. It also has advantage over the facility-based treatment as there are poor cultural acceptances, chances of hospital-acquired infections and high relapse rates in the facility based treatment [11].

The effectiveness of CMAM programme is primarily measured as proportion of children cured or recovered, which is defined as a child achieving WHZ ≥ -2 and/or MUAC ≥125 mm and through other measures like the rate of weight gain, time to recovery, defaulter rate, relapse rate and mortality rate. Sustainability of recovery has long been ignored in the outcomes of SAM treatment as primary focus of treatment was on reducing mortality from SAM. However, in recent years, implementers have begun to consider the extent to which treatment enables a recovery in both short and long terms [12].

SAM is clinically characterized by loss of muscle and fat tissue, reflecting an inadequate intake of energy and protein relative to body requirements. It also has a broader range of pathophysiological changes which are associated with reduced function of the body, specifically metabolic function, oxidative stress, cardiac function, hepatic function, enteropathy, renal and brain function [13]. Nutritional recovery of young children is typically gauged by short-term weight gain. However, an increase in weight does not necessarily reflect an increase in fat-free mass (FFM) as weight gain following severe illness has been shown to be secondary to gains in fat mass (FM) rather than FFM. Despite accounting for height and weight, body mass index (BMI) also does not accurately reflect FFM, as individuals with similar BMI can have significant variations in FFM [14]. Hence, it becomes important to additionally assess body composition (such as FM, FFM and total body water) to evaluate whether these parameters among rehabilitated SAM children are comparable with the normal children however, this has been done in very limited studies. Skinfold thickness and assessment of body composition by bio-electrical impedance analysis (BIA) is a better indicator of energy reserves. Skinfold measurements- tricep and sub-scapular, arm muscle area (AMA) and arm fat area (AFA) provide a simple, easy, and quick yet highly informative assessment of fatness in most patients. The analysis of body composition by BIA method produces estimates of total body water (TBW), fat-free mass (FFM), and fat mass (FM) by measuring the resistance of the body as conductor to a very small alternating electrical current or by administration of radioactive isotope [15, 16]. Application of the combination of these methods may reduce the likelihood of misdiagnosis of high or low lean mass [17].

Most of the evidence on the effectiveness of the CMAM programme comes from observational studies conducted in emergency settings in Africa [18]. Also, these evidence of effectiveness are largely gauged through anthropometric parameters and not through body composition. While CMAM has been adopted by many countries worldwide, India is yet to have a formal national guideline for CMAM strategy due to lack of consensus on using RUTF in the programme. There are a few pilot studies on CMAM conducted in India using the WHO recommended RUTF for assessing efficacy of the community based programmes. Such studies with RUTF achieved recovery rates ranging from 46.7 to 57.4% [19–23] however, beyond anthropometric parameters these studies did not report any finding related to the child’s body composition.

## Methods

### Rationale for the study

Currently in India, some of the state governments have decided to conduct the community-based SAM treatment programme, hereinafter called CSAM, using a variety of locally produced nutrient dense food supplements, which has different energy density and nutrient composition. Locally produced nutrient dense food supplements have been chosen by state governments considering factors such as cost, availability and ease of programming. Equally, the CSAM protocol to be used in each state’s CSAM program varies. Little is known about the effectiveness and quality of recovery of children using these locally produced nutrient dense food supplements to be used under these programmes. Therefore, we propose to assess and compare the effectiveness of these programmes at two levels across five states. First, at the programme level through an assessment of programme outcomes in terms of cure rate, weight gain, relapse and mortality, and second, at the level of the individual child by assessing changes in body composition of the child over the course of treatment. This study also meets the recommendations of the WHO expert group meeting, which agreed that the demonstration of the effectiveness of a food for the treatment of acute malnutrition should not be based solely on anthropometric parameters [24].

There is a need to understand the extent to which recovery as per anthropometric and body composition parameters is achieved in each state compared to the state with no CSAM intervention (comparison state). The policy implications of the proposed study are significant in that they will provide evidence on performance of locally produced nutrient dense food supplements and currently implemented CSAM protocols in different states which will aid states in their efforts to strengthen their existing CSAM protocols and alternative food supplements.

### Intervention strategy

In general, the State Governments of Telangana, Madhya Pradesh, Odisha and Chhattisgarh will implement community-based management of SAM (CSAM) programme adapted from the WHO guidelines, in addition to the existing government nutrition promotion interventions. The approach will incorporate active and/or passive case-finding and screening for severe wasting by government frontline nutrition and health workers. For uniformity, children with WHZ < -3 will be categorized as experiencing SAM. SAM children will further undergo medical examinations and appetite test to identify complicated and uncomplicated cases. All complicated cases will be referred to the nearest nutritional rehabilitation centres (NRCs) for in-patient management. All uncomplicated cases will be enrolled in the CSAM programme for their community-based management in *anganwadi centres*. While the program’s primary focus is on treatment of children who are severely wasted, key activities also include regular follow up of all enrolled children at the *anganwadi centers*, counseling of caregivers on infant and young child feeding (IYCF), safe water, sanitation and hygiene practices, family planning and child care practices, promotion of vaccinations, appropriate referrals of sick children to NRCs or public health facilities and post discharge follow up. SAM children enrolled in the programme will also be given locally produced nutrient dense food supplements, antibiotics, anthelminthic and micronutrient supplements. Key aspects of the state specific CSAM programme are presented in Table 1. Detailed description of the state specific CSAM protocols and nutritional content of each locally produced nutrient dense food supplement is provided in Additional file 1. In the comparison area, where no CSAM intervention is being implemented, SAM children identified and enrolled in the study will receive the existing government nutrition promotion interventions. The existing program includes provision of double quantity of Take Home Ration (THR)^1^ for severely underweight children (which covers most of SAM children) and identification and referral of SAM children to Malnutrition Treatment Centres (MTCs).

**Table 1:** Key aspects of the CSAM protocol that vary per state assessed

States	Screening & Identification	Admission criteria	Maximum treatment duration	Discharge criteria	Nutrient dense food	Post-discharge Follow up
Telangana	Active screening- once in every quarter Passive screening on all MCH contacts	SAM identification by WHZ < -3SD	16 weeks	WHZ ≥ -2SD	Balamrutam+ under ICDS provision (composition- whole wheat, Bengal gram, roasted groundnut, Jaggery, vegetable oil and added vitamin & minerals) *477 Kcal and 11 g protein per 100 g*	Monthly follow up for 6 months
Madhya Pradesh	Active screening- once in every month Passive screening on VHSND/Health Sub-centre& all MCH contacts	SAM identification by WHZ < -3SD	12 weeks	WHZ ≥ −2SD	Take Home Ration (THR) under ICDS provision by MP Agro. State THR is a mix of cereals, pulses, oil, sugar, milk powder and soya bean. *440 Kcal and 17 g protein per 100 g*	Monthly follow up for 3 months
Chhattisgarh	Active screening- once in every quarter Passive screening on VHSND/Health Sub-centre& all MCH contacts	SAM identification by WHZ < -3SD	16 weeks	WHZ ≥ -2SD	ICDS based modified THR (wheat, soybean, chickpea, sugar, vegetable oil, groundnut, finger millet). *410 Kcal and 12.75 g protein per 100 g*	1st, 3^rd^and 6th month
Odisha	Active screening- once in every quarter Passive screening on VHSND/Health Sub-centre& all MCH contacts	SAM identification by WHZ < -3SD	16 weeks	WHZ ≥ -2SD	Augmented ICDS THR (additional oil, groundnut and whole milk powder). *462 Kcal and 16 g protein per 100 g* 1 egg daily in addition to THR	1st, 3rd and 6th month

### Study aim

We aim to evaluate the effectiveness of different state specific CSAM interventions to assess their impact on anthropometry and body composition among SAM children.

### Research questions

#### Primary research question

What is the effectiveness of the CSAM programmes in four states compared to the comparison/control state among children aged 6–59 months with uncomplicated SAM in rural India?

#### Secondary research questions

1. Do state specific CSAM programmes help recovery of a SAM child, as defined by body composition parameters such as fat mass by skinfold thickness and fat free mass, body water by BIA, in each of the four intervention states?
2. What is the relationship between anthropometry and body composition measures in a child suffering from SAM, in each of the five states?
3. Do state specific CSAM programme provide better outcomes in terms of recovery, defaulting, death, length of stay and average weekly weight gain, compared to existing evidence from India?
4. Which of the CSAM programme provides best rate of recovery 6 months after exit from the programme, defined by the relapse and mortality rates?

### Study design and setting

This multi centric longitudinal quasi-experimental study with a comparison area will take place in two blocks each in Gadwal (state Telengana), Hoshangabad (state Madhya Pradesh), Rajnandgaon (state Chhattisgarh), Nabrangpur (state Odisha) and Latehar (state Jharkhand) districts. More than 80% population in these districts reside in rural areas with more than one-third of the households being poor [25]. The prevalence of wasting and severe wasting among children in these districts are very high. Wasting among children under five in these districts are 18.6, 29.6, 17.2, 36.0 and 29.0% respectively and severe wasting prevalence is5.1, 10.6, 6.2, 11.6 and 10.7% respectively [4]. From each of the four intervention districts, two study Blocks will be purposively selected where either the CSAM intervention is currently being implemented or have been planned by the respective state governments. Similarly, two study Blocks from Latehar district of the state Jharkhand will be purposively selected as comparison area, where no CSAM intervention is being implemented.

The study will be led by Kalawati Saran Children’s Hospital (KSCH), New Delhi and supported by Children’s Investment Fund Foundation (CIFF) through UNICEF, India. KSCH will partner with Indian Council of Medical Research-National Institute of Nutrition (ICMR-NIN), Telangana, All India Institute of Medical Science (AIIMS) in Madhya Pradesh and Chhattisgarh, National Health Mission (NHM) of Odisha and Rajendra Institute of Medical Science (RIMS), Jharkhand, who will administer and manage the study in the respective states.

### Sample size

The primary outcome of the study is cure rate at 12 weeks (in Chhattisgarh) or 16 weeks (in Telangana, Madhya Pradesh and Odisha) compared to the comparison area. Evidence on CMAM interventions in India testing its efficacy have shown cure rates ranging from 46.7 to 57.4% [19–23]. Another study from India found cure rate among 25–30% children with minimal community based interventions [26]. Hence for the sample size calculation, we therefore chose the expected cure rate with the proposed CSAM interventions of 50% and expected cure rate with existing government nutritional interventions of 25% to estimate the effect size of 25% (50–25%). The sample in each state will comprise of an equal proportion of children from the two age groups (6–23 months and 24–59 months). Using the significance level of 5%, with 80% power to detect the effect size of 25%, a design effect of 1.5 and accounting for 20% attrition due to loss of follow up and mortality, the sample size calculated for each age group of children is 100. Hence, the proposed sample size for the two age-groups of children in each state is 200 children. In total, the study will enroll 1000 children in all the five study states.

### Selection of anganwadi centres

In each participating state, 100 *anganwadi centres* from the two study Blocks will be selected using probability proportion to size (PPS) sampling technique.

### Selection of study samples

In each of the CSAM intervention states, 200 SAM children aged 6–59 months identified using WHZ < -3, who will be enrolled in the CSAM programme and who do not have any medical complication and pass the appetite test will be enrolled in the study. Similarly, in the comparison state, 200 SAM children aged 6–59 months will be identified using WHZ < -3 by the Field Investigators and enrolled in the study, who do not have any medical complication and pass the appetite test. Children identified between October’ 2020 and December’ 2020 will be enrolled in the study.

We will exclude SAM children with presence of emergency signs, edema, high fever, cough with fast breathing and known secondary SAM cases like neurological diseases such as cerebral palsy, TB, HIV congenital heart defects, congenital structural defects and cleft palate. However, if any child enrolled in the study develops any medical complication later during the course of the study, s/he will be referred to inpatient care but continued to be part of the study.

The sub-set of children (first 50% of the enrolled children i.e. 100 children in each state) with weight at enrollment > 5 kg will be selected for body composition, as the BIA machine can only give accurate measurement for children with weight > 5 kg. So in total, 500 children will be selected for studying their body composition in the five study states.

For identifying eligible study participants in the CSAM intervention states, SAM children to be identified and enrolled by the frontline workers according to the identification criteria of the state specific CSAM programme from each of these selected *anganwadi centres*, will be rechecked by field investigators within 72 h and those children who will qualify on the basis of inclusion and exclusion criteria of the study, will be enrolled in the study. Similarly, in the comparison state, Field Investigators will identify eligible study participants and enroll in the study. Field Investigators will then approach caregivers of the children to explain the study and seek their written consent for participation. They will also be asked for permission to follow-up their children during the treatment phase and for 6 months’ post discharge in order to measure the impact of the interventions.

### Data collection, cleaning and storage

In the intervention states, the children enrolled in the study will be managed according to the state specific CSAM guidelines and locally produced nutrient dense food supplements. The study will assess the effectiveness of the CSAM programmes in terms of recovery of these children compared to the comparison arm. This will be assessed using anthropometric (length/height and weight) measurements and skinfold thickness (triceps and sub-scapular) to be taken on admission, at sixth week and at discharge by the trained field investigator. For anthropometric measurements, a digital weighing machine with a sensitivity of 10 g to measure weight and a wooden infant-cum-stadiometer to measure length/ height will be used. Length and height will be measured to the nearest 0.1 cm, weight to the nearest 0.01 kg and skinfold thickness to the nearest 0.1 cm. Weighing equipment will be calibrated using standard calibration weights and infant-cum-stadiometer will be calibrated with calibration rods. The skinfold caliper will also be calibrated daily. Before doing the data collection work, all Field Investigators will be trained and also complete anthropometric standardization exercises where their anthropometric measurement skills will be assessed for thoroughness, accuracy and precision. To assess the accuracy and precision of each Field Investigator, their measures will be compared to those of the master trainers, who will be considered the gold standard. Intra-observer technical error of measurement (TEM) and reliability will be calculated using the difference between the two measures of the Field Investigators. Inter-observer TEM and reliability will be calculated by comparing the Field Investigators two measures to the trainer’s measure [27].

Also, during admission and at discharge, the Field Investigator will administer the questionnaire for capturing child details and incidences of morbidity. Additionally, during admission, data on availability of drinking and sanitation facilities and other household level socio-economic information will be collected. Also,24-h dietary recall, food frequency and food consumption questionnaires will be administered to these children on admission, at sixth week and at discharge to study food consumed by the children, food frequency and compliance of locally produced nutrient dense food supplements provided to the children as part of the intervention. Futhermore, we will also ascertain cause of death of any enrolled child died during the study period.

Besides, first 100 enrolled children in that state who qualifies for body composition measurement i.e. weight on admission > 5 kg, will also be measured for body composition parameters using BIA machine (InBody S10) on admission and at discharge.

Post discharge, the children will be followed up on a monthly basis for anthropometry until 6 months by the field investigators. Additionally, skinfold thickness and body composition assessment of all children will also be repeated at sixth month post discharge. A detailed description of data collection process is shown in Table 2.

**Table 2:** Overview of all measurements that will be taken from children

Parameters	Method	Admission/ Baseline	6th Week	12th/16thweek/Exit/Discharge	Post-discharge Follow up^b^
All children enrolled:					
Child details, and history of illness	Questionnaire	**✓**		**✓**	
Drinking water and sanitation facilities and other household socio-economic status		**✓**			
Weight, Recumbent length/ Height,	Digital weighing machine, Infantometer/ Stadiometer	**✓**	**✓**	**✓**	✓
Skinfold Thickness (triceps & subscapular skinfold)	Holtain Skinfold Caliper	**✓**	**✓**	**✓**	✓^c^
Dietary assessment (24-h dietary recall & food frequency) and Consumption of locally produced nutrient dense food	Observation and questionnaire	**✓**^**a**^	**✓**	**✓**	
Body composition; For enrolled sub-sample of children (weight > 5 kg)					
Body fat mass	BIA machine	✓		✓	
Body lean mass					✓^c^

^a^At baseline only the 24-h dietary recall & food frequency assessment will be done

^b^Post discharge, all children will be prospectively followed up for anthropometric measurements on a monthly basis till 6 months by the field investigators

^c^Additionally, skinfold thickness and body composition assessment of all children will also be repeated at 6^th^ month post discharge

We will use mobile phones programmed using the Kobocollect platform for data collection. Every child will be given a unique number which will be used to link the database of the child to be collected at different time points. The monitoring team will check the number of interviews completed and type of data collected on regular basis in order to promptly identify and address possible problems. The data will be downloaded regularly and checked for errors using automated do files and manual inspections. The data will be stored and backed up every 2 weeks on a server.

### Quality control

Quality control visits will be conducted by an independent monitoring team constituted from KSCH using a standardized checklist. Monitoring team will do data quality checks of atleast 10% of enrolled children.

### Data analysis

Final analysis will include all children enrolled in the study. Appropriate quantitative analyses will be used including descriptive statistics (proportion, percentage, mean or median), comparison of difference in proportion for binary outcome using Pearson chi-square and comparison of average change for continuous variable using t-tests. Recovery will be compared for effectiveness of state specific CSAM protocols and locally produced nutrient dense food supplements in four intervention states in comparison to the comparison state. Cox regression method will also be used for this analysis by defining recovery as event, as it can utilize the entire sample size including defaulters or loss to follow up. Other parameters including skinfold, defaulting, death, length of stay, average weekly weight gain and height gain will also be compared between the intervention states and the control state. Subgroup analysis will be done for comparing recovery among children aged 6–23 months and 24–59 months between intervention states and control state. For the sub-sample of body composition, change in fat mass, fat free mass and body water will be compared between the four intervention states and the comparison state. A linear mixed model will be developed for different outcomes (recovery, weight gain, and body composition parameters) adjusting for various random and fixed effects across all sites adjusted for differences at baseline.

### Process evaluation

A process evaluation will document the design, implementation and mechanisms of the CSAM intervention in the study states to enable replication and scale up. It will use data from the CSAM planning and monitoring document and forms to capture compliance, challenges and learning. Furthermore, semi-structured interviews with state, district and block officials, frontline workers and caregivers and focus group discussions with small family groups and frontline workers will be conducted to document perceptions of the intervention and its effects.

### Cost effectiveness analysis

We will conduct cost and cost-effectiveness analyses to guide policy decisions and inform any subsequent scale up. We will estimate the total and incremental costs of the CSAM intervention in each of the four intervention study states from the provider’s perspective. The costs of delivering the CSAM interventions both at the community and the facility levels will be collected retrospectively from the accounts of government and other partnering institutions, using a standardised data capture tool designed for this purpose in Excel. This will also be combined with key informant interviews and available financial data on the average unit cost of delivering those services. Time use study will be conducted with frontline workers and other key implementers to value their time spent in the CSAM intervention. Cost of any donated resource to the CSAM intervention will also be included in the analysis. All costs will be adjusted for inflation using the Indian Consumer Price Index (CPI) and will be presented in Indian Rupees and 2021 International Dollars. Incremental cost-effectiveness will be measured in relation to the status quo alternative i.e. doing nothing in addition to the current ongoing activities as part of government nutrition promotion initiatives. Incremental cost-effectiveness ratios (ICERs) will be calculated for the primary outcome measures (i.e. SAM cases treated and cured/recovered) and selected secondary outcome measures such as cases of child mortality averted, life-years saved and disability adjusted life years (DALYs) averted. Sensitivity analyses will be carried out to assess the robustness of results.

## Discussion

Different state governments have contextualized, designed and developed operational CSAM protocols for implementing their state specific CSAM strategies to be manageable within their existing NHM and ICDS systems. The study results will be generalisable to rural areas of India with a high burden of childhood wasting and underserved community. There are a large number of high-burden districts and Blocks in India where prevalence of SAM among children is very high. If the CSAM strategies of different states improve the study’s primary and/or secondary outcomes, the intervention strategies can be scaled up to all or parts of the high- burden districts through NHM and ICDS systems. These intervention strategies will also enable the health and ICDS systems to work in collaboration with each other at the village level through their existing frontline workers.

### Trial timeline and status

The trial is planned for a duration of 12 months (1st December 2020 – 30th November 2021) and is currently recruiting participants.

## Supplementary Information

**Additional file 1.** Overview of the state specific CSAM protocols for management of uncomplicated SAM.

## Data Availability

Not Applicable.

## Abbreviations

SAM: Severe Acute Malnutrition
CMAM: Community-based Management of Acute Malnutrition
WHO: World Health Organisation
CSAM: Community-based Management of Severe Acute Malnutrition
AWC: Anganwadi Centre
WHZ: Weight for Height Z-score
BIA: Bio-electrical Impedance Analysis
TBW: Total Body Water
FFM: Fat Free Mass
FM: Fat Mass
MUAC: Mid Upper Arm Circumference
RUTF: Ready to Use Therapeutic Food
BMI: Body Mass Index
AMA: Arm Muscle Area
AFA: Arm Fat Area
NRC: Nutritional Rehabilitation Centre
IYCF: Infant and Young Child Feeding
THR: Take Home Ration
MTC: Malnutrition Treatment Centre
KSCH: Kalawati Saran Children’s Hospital
CIFF: Children’s Investment Fund Foundation
UNICEF: United Nations Children’s Fund
AIIMS: All India Institute of Medical Sciences
NHM: National Health Mission
RIMS: Rajendra Institute of Medical Sciences
ICMR-NIN: Indian Council of Medical Research-National Institute of Nutrition
PPS: Probability Proportional to Size
TB: Tuberculosis
HIV: Human Immunodeficiency Virus
TEM: Technical Error of Measurement
CPI: Consumer Price Index
ICER: Incremental Cost Effectiveness Ratio
DALY: Disability Adjusted Life Year
ICDS: Integrated Child Development Services

## Footnotes

1 THR contains wheat, roasted groundnut, pulses, jaggery, potato and chickpea. Each 310 g meal provides approximately 938 kcal of energy and 33.5 g of protein.
