# Supplementary material for: Effectiveness of community-based treatment programs for treatment of uncomplicated severe acute malnourished children aged 6–59 months using locally produced nutrient dense foods: protocol for a multicentric longitudinal quasi-experimental study
Source: BMC Nutr. 2021 Dec 15;7:85. doi: 10.1186/s40795-021-00489-1 (PMC8672603; doi:10.1186/s40795-021-00489-1)
Supplement: Supplementary file 1 — Additional file 1. Overview of the state specific CSAM protocols for management of uncomplicated SAM. [file 40795_2021_489_MOESM1_ESM.docx]

| **Additional file 1: Overview of the state specific CSAM protocols for management of uncomplicated SAM** | |
| --- | --- |
| **Telangana** | |
| **Screening of children with SAM** | Screening of SAM cases will be done in the community using weight for height Z score and bilateral pitting edema  ***Active Screening:***  **Who:** Acredited Social Health Activitist (ASHA) and Anganwadi Teacher (AWT) will conduct house to house screening of all children aged 6-59 months in their village.  **When:** once in every quarter  ***Passive Screening:***  **Who:** Frontline workers [Auxilary Nurse Midwife (ANM), Mitanin and AWT] will conduct screening of all children aged 6-59 months in her village during all maternal and child health (MCH) contacts which include: Sick child consultation at the Sub-centre, Nutrition and Health Day (NHD), Immunization Days, Biannual Vitamin A rounds etc.  Children with bilateral pitting edema will be referred to the nearest NRC for their treatment. All children with WHZ<-3 will be mobilsed to NHD of that village for medical assessment and appetite test by the ANM. Complicated SAM cases will be referred to the nearest NRC and uncomplicated cases will be enrolled in the CSAM programme. |
| **Locally Produced Nutrient Dense Food Supplement** | Nutritional care will be provided for Children through Balamarutham + (nutrient dense product developed by NIN).  Its nutrient composition will be whole wheat 35 g, green gram/bengalgram dal 20g, ground nut 15g – roasted and grinded separately into powder. Add jaggery 30g and 10ml vegetable oil to make the mixture ready to eat. Vitamins and minerals may be added at point of use. It provides 477 Kcal and 11 g protein per 100 g.  The Balamarutham + can be kept safely for several days after the package is opened provided it is protected from flies, insects and rodents. The amount of food given to the child should be sufficient to take care of the caloric requirement of 175- 200 kcal per kilogram of body weight per day.  The Balamarutham + can be augmented with milk, nuts, ghee /oil to make it more energy dense and diversified. |
| **Medicines and other supplements** | **Amoxicillin DT** (125mg)- First dose on enrolment and then for home (two times daily for five days) for  Child 1 to 3.5 months-1 tablet twice daily for 5 days,  Child 4 to 12 months-1.5 tablets twice daily for 5 days,  Child 13 to 28 months-2 tablets twice daily for 5 days,  Child 29 to 59 months-2.5 tablets twice daily for 5 days,  **Albendazole** (5ml=200mg) on second visit, for  Child <12 months-none,  Child 12-23 months-5ml,  Child ≥24 months-10ml.  **Folic acid**-1 dose of 5mg on day 1.  **Multivitamin** daily 5ml for 90 days  **Vitamin A**- One dose on admission to the SAM child if not given during last 1 month and also if there is next biannual round of vitamin A upcoming with next 1 month.  For the child 6 to 12 months-100000 International Unit (IU)/1ml  For the child >12 months-200000 IU/2 ml  **IFA-**1ml IFA syrup biweekly for 4 months having 20mg elemental iron and 100mcg folic acid  **Zinc**-2.5ml/child/day for 14 days if weight of the child is <5kg and 5ml/child/day for 14 days if weight of the child is >5kg. |
| **Follow up during treatment** | The SAM child will be followed up for the maximum duration of 16 weeks.  **Follow up:** weekly follow up at the AWC.  **Activities to be undertaken during follow up visit:** Weight, height/length record and assessment for edema, medical check-up and medical history (illness in the previous fortnight) by ANMs with support from AWT, the child receives drugs and Balamrutam+, individual counselling and health and nutrition education in groups by AWTs |
| **Discharge criteria** | **Discharged and cured:** Child who has attained WHZ ≥ -2SD for two consecutive follo ups within the time frame of 16 weeks from admission  **Not recovered:** Child who do not attained WHZ ≥ -2SD within the time frame of 16 weeks from admission  **Referral:** All cases in which medical complications developed / secondary causes of malnutrition detected and referred for appropriate treatment  **Defaulter:** Children who left the programme before reaching the discharge criteria or those who were absent for two consecutive visits  **Died:** Child who died from any cause while treatment in the programme. |
| **Follow up post discharge** | The Child will be followed up for six months post discharge from the program.  **Follow up:** monthly follow up at the AWC.  **Activities to be undertaken during follow up visit:** Weight, height/length record and assessment for edema, medical check-up and medical history (illness in the previous fortnight) by ANMs with support from AWT, individual counselling and health and nutrition education in groups by AWTs |
| **Madhya Pradesh** | |
| **Screening of children with SAM** | Screening of SAM cases will be done in the community using weight for height Z score and bilateral pitting edema  ***Active Screening:***  **Who:** ASHA and Anganwadi Worker (AWW) will conduct house to house screening of all children aged 6-59 months in their village.  **When:** once in every quarter  ***Passive Screening:***  **Who:** Frontline workers (ANM, ASHA and AWW) will conduct screening of all children aged 6-59 months in her village during all MCH contacts which include: Sick child consultation at the Sub-centre, VHSND, Immunization Days, Biannual Vitamin A rounds and Routine Growth Monitoring and Promotion activity or THR distribution day at the AWC.  Children with bilateral pitting edema will be referred to the nearest NRC for their treatment. All children with WHZ<-3 will be mobilsed to VHSND of that village for medical assessment and appetite test by the ANM. Complicated SAM cases will be referred to the nearest NRC and uncomplicated cases will be enrolled in the CSAM programme. |
| **Locally Produced Nutrient Dense Food Supplement** | The child will be provided energy dense food supplement that provides approximately 440 kcal of energy and 17g protein per 100 grams. It consists of cereals, pulses, oil, sugar, milk powder and soya bean.  Provision of THR for 6-59 months under the CSAM programme as per the weight of the child.  Child 3.5-5.7 kg - 2 THR packets per week (600gm/packet),  Child 5.8-8.0 kg - 3 THR packets per week,  Child 8.1-10.4 kg - 4 THR packets per week and  Child ≥10.5 kg - 5 THR packets per week. |
| **Medicines and other supplements** | **Amoxicillin DT** (125ml/5ml)- First dose on enrolment and then for home (two times daily for five days) for  Child 3 to 5kg-2.5ml twice daily for 5 days,  Child 5 to 7kg-5ml twice daily for 5 days,  Child 7-9kg-7.5ml twice daily for 5 days,  Child 9 to 11kg-10ml twice daily for 5 days,  **Albendazole** (5ml=200mg) on first visit, for  Child <12 months-none,  Child 12-23 months-5ml,  Child ≥24 months-10ml.  **Folic acid**-1 dose of 5mg on day 1.  **Iron syrup**-1 ml twice in a week  **Multivitamin** daily 5ml for 90 days |
| **Follow up during treatment** | The SAM child will be followed up for the maximum duration of 12 weeks.  **Follow up:** weekly follow up at the AWC.  **Activities to be undertaken during follow up visit:** Weight, height/length record and assessment for edema, medical check-up and medical history (illness in the previous fortnight) by ANMs with support from AWW, the child receives drugs and energy dense food supplement, individual counselling and health and nutrition education in groups by AWWs |
| **Discharge criteria** | **Discharged and cured:** Child who has attained WHZ ≥ -2SD for two consecutive follow ups within the time frame of 12 weeks from admission  **Not recovered:** Child who do not attained WHZ ≥ -2SD within the time frame of 12 weeks from admission  **Referral:** All cases in which medical complications developed / secondary causes of malnutrition detected and referred for appropriate treatment  **Defaulter:** Children who left the programme before reaching the discharge criteria or those who were absent for three consecutive visits  **Died:** Child who died from any cause while treatment in the programme. |
| **Follow up post discharge** | The Child will be followed up for three months post discharge from the program.  **Follow up:** Monthly for three months at the AWC.  **Activities to be undertaken during follow up visit:** Weight, height/length record and assessment for edema, medical check-up and medical history (illness in the previous fortnight) by ANMs with support from AWW, individual counselling and health and nutrition education in groups by AWWs. |
| **Chhattisgarh** | |
| **Screening of children with SAM** | Screening of SAM cases will be done in the community using weight for height Z score and bilateral pitting edema  ***Active Screening:***  **Who:** Mitanin and Anganwadi Worker (AWW) will conduct house to house screening of all children aged 6-59 months in their village.  **When:** once in every quarter  ***Passive Screening:***  **Who:** Frontline workers (ANM, Mitanin and AWW) will conduct screening of all children aged 6-59 months in her village during all MCH contacts which include: Sick child consultation at the Sub-centre, Village Health Sanitation and Nutrition Days (VHSND), Immunization Days, Biannual Vitamin A rounds during Bal Swasthya Poshan Maah (BSPM) and Routine Growth Monitoring and Promotion activity, during Community Based Events (CBEs) or Take Home Ration (THR) distribution day at the AWC.  Children with bilateral pitting edema will be referred to the nearest NRC for their treatment. All children with WHZ<-3 will be mobilsed to VHSND of that village for medical assessment and appetite test by the ANM. Complicated SAM cases will be referred to the nearest NRC and uncomplicated cases will be enrolled in the CSAM programme. |
| **Locally Produced Nutrient Dense Food Supplement** | The child will be provided energy dense food supplement that provides approximately 410 Kcal and 12.75 g protein per 100 g. This is a combination of four food group items namely cereals, pulses, oil and sugars (wheat 30g, soyabean 10g, chickpea 20g, sugar 27g, vegetable oil 5g, groundnuts 5g, finger millet 3g).  The state’s plan to provide energy dense food supplement per day to different age-groups of children through its ICDS system is given below:   1. For children between 6-36 months: 200 g/day (821 Kcal and 25.5 g Protein) and 2. For children between 36- 59 months: 75 g/ day (307 Kcal and 9.55 g protein) and a hot cooked meal. |
| **Medicines and other supplements** | **Amoxicillin DT** (125mg)- First dose on enrolment and then for home (two times daily for five days) for  Child 1 to 3.5 months-1 tablet twice daily for 5 days,  Child 4 to 12 months-1.5 tablets twice daily for 5 days,  Child 13 to 28 months-2 tablets twice daily for 5 days,  Child 29 to 59 months-2.5 tablets twice daily for 5 days,  **Albendazole** (5ml=200mg) on second visit, for  Child <12 months-none,  Child 12-23 months-5ml,  Child ≥24 months-10ml.  **Folic acid**-1 dose of 5mg on day 1.  **Multivitamin** daily 5ml for 90 days  **Vitamin A**- One dose on admission to the SAM child if not given during last one month and also if there is next biannual round of vitamin A upcoming with next one month.  For the child 6 to 12 months-100000 International Unit (IU)/1ml  For the child >12 months-200000 IU/2 ml  **IFA-**1ml IFA syrup biweekly for 4 months having 20mg elemental iron and 100mcg folic acid  **Zinc**-2.5ml/child/day for 14 days if weight of the child is <5kg and 5ml/child/day for 14 days if weight of the child is >5kg. |
| **Follow up during treatment** | The SAM child will be followed up for the maximum duration of 16 weeks.  **Follow up:** fortnightly follow up at the AWC.  **Activities to be undertaken during follow up visit:** Weight, height/length record and assessment for edema, medical check-up and medical history (illness in the previous fortnight) by ANMs with support from AWW, the child receives drugs and energy dense food supplement, individual counselling and health and nutrition education in groups by AWWs |
| **Discharge criteria** | **Discharged and cured:** Child who has attained WHZ ≥ -2SD for two consecutive follow ups within the time frame of 16 weeks from admission  **Not recovered:** Child who do not attained WHZ ≥ -2SD within the time frame of 16 weeks from admission  **Referral:** All cases in which medical complications developed / secondary causes of malnutrition detected and referred for appropriate treatment  **Defaulter:** Children who left the programme before reaching the discharge criteria or those who were absent for two consecutive visits  **Died:** Child who died from any cause while treatment in the programme. |
| **Follow up post discharge** | The Child will be followed up for six months post discharge from the program.  **Follow up:** 1^st^, 3^rd^ and 6^th^ month at the AWC during VHSND.  **Activities to be undertaken during follow up visit:** Weight, height/length record and assessment for edema, medical check-up and medical history (illness in the previous fortnight) by ANMs with support from AWW, individual counselling and health and nutrition education in groups by AWWs |
| **Odisha** | |
| **Screening of children with SAM** | Screening of SAM cases will be done in the community using weight for height Z score and bilateral pitting edema  ***Active Screening:***  **Who:** ASHA and Anganwadi Worker (AWW) will conduct house to house screening of all children aged 6-59 months in their village.  **When:** once in every quarter  ***Passive Screening:***  **Who:** Frontline workers (ANM, ASHA and AWW) will conduct screening of all children aged 6-59 months in her village during all MCH contacts which include: Sick child consultation at the Sub-centre, VHSND etc.  Children with bilateral pitting edema will be referred to the nearest NRC for their treatment. All children with WHZ<-3 will be mobilsed to VHSND of that village for medical assessment and appetite test by the ANM. Complicated SAM cases will be referred to the nearest NRC and uncomplicated cases will be enrolled in the CSAM programme. |
| **Locally Produced Nutrient Dense Food Supplement** | In the Supplementary Nutrition Programme (SNP) of ICDS in the state, there is a provision of Take Home Ration (THR), morning snacks and hot cooked meals for severely underweight children within the age group of 6 months to 6 years. Complete SNP diet is diversified and contains cereals and millets, pulses, oilseeds, oils, sugar, fruits, vegetables and eggs which provide 1125.5 Kcal energy and 33.6 gm protein on daily basis to children aged 6 months to 3 years, whereas 1005 kcal energy and 27 gm protein on daily basis to children within the age group of 3 to 6 years receive. One egg will also be provided daily in addition to the THR.  To fulfil the additional nutritional requirement of SAM children, existing THR will be augmented by adding additional oil, groundnut and whole milk powder to make them more energy dense and diversified. |
| **Medicines and other supplements** | **Amoxicillin DT** (125mg)- First dose on enrolment and then for home (two times daily for five days) for  Child 1 to 3.5 months-1 tablet twice daily for 5 days,  Child 4 to 12 months-1.5 tablets twice daily for 5 days,  Child 13 to 28 months-2 tablets twice daily for 5 days,  Child 29 to 59 months-2.5 tablets twice daily for 5 days,  **Albendazole** (5ml=200mg) on second visit, for  Child <12 months-none,  Child 12-23 months-5ml,  Child ≥24 months-10ml.  **Folic acid**-1 dose of 5mg on day 1.  **Multivitamin** daily 5ml for 90 days  **Vitamin A**- One dose on admission to the SAM child if not given during last one month and also if there is next biannual round of vitamin A upcoming with next one month.  For the child 6 to 12 months-100000 International Unit (IU)/1ml  For the child >12 months-200000 IU/2 ml  **IFA-**1ml IFA syrup biweekly for 4 months having 20mg elemental iron and 100mcg folic acid  **Zinc**-2.5ml/child/day for 14 days if weight of the child is <5kg and 5ml/child/day for 14 days if weight of the child is >5kg. |
| **Follow up during treatment** | The SAM child will be followed up for the maximum duration of 16 weeks.  **Follow up:** fortnightly follow up at the AWC.  **Activities to be undertaken during follow up visit:** Weight, height/length record and assessment for edema, medical check-up and medical history (illness in the previous fortnight) by ANMs with support from AWW, the child receives drugs and energy dense food supplement, individual counselling and health and nutrition education in groups by AWWs**.** |
| **Discharge criteria** | **Discharged and cured:** Child who has attained WHZ ≥ -2SD for two consecutive follow ups within the time frame of 16 weeks from admission  **Not recovered:** Child who do not attained WHZ ≥ -2SD within the time frame of 16 weeks from admission  **Referral:** All cases in which medical complications developed / secondary causes of malnutrition detected and referred for appropriate treatment  **Defaulter:** Children who left the programme before reaching the discharge criteria or those who were absent for two consecutive visits  **Died:** Child who died from any cause while treatment in the programme. |
| **Follow up post discharge** | The Child will be followed up for six months post discharge from the program.  **Follow up:** 1^st^, 3^rd^ and 6^th^ month at the AWC during VHSND.  **Activities to be undertaken during follow up visit:** Weight, height/length record and assessment for edema, medical check-up and medical history (illness in the previous fortnight) by ANMs with support from AWW, individual counselling and health and nutrition education in groups by AWWs. |
